# Modern contraceptive use among currently married non-pregnant women (aged 15–49 years) in West Bengal, India: a reflection from NFHS-5

**DOI:** 10.1186/s40834-024-00322-7

**Published:** 2024-11-29

**Authors:** Susanta Sen, Amit Banerjee, Asif Ali, Namita Chakma

**Affiliations:** Department of Geography, The University of Burdwan, 713104 Burdwan, West Bengal India

**Keywords:** Modern contraceptive use, Spatial variation, Socio-demographic factors, NFHS-5, West Bengal

## Abstract

**Background:**

There is a dearth of research on modern family planning practices in high focus states like West Bengal in India with diverse population groups and distinct health needs. Thus, this study aims to investigate the latest picture of modern contraceptive use and its associated socio-demographic factors among currently married non-pregnant of reproductive aged (15–49 years) women in West Bengal.

**Methods:**

The study is based on secondary data, collected from the fifth round of the National Family Health Survey (NFHS-5; 2019-20). A total of 15,841 currently married non-pregnant women were included into this study. With the overarching goal of understanding the determinants and patterns of modern contraceptive use, the study employed a combination of bi-variate and multivariate analyses, including logistic regression.

**Results:**

The results reveal that female sterilization is the most common modern contraceptive method across the state. Contraceptive use varies by district, from 43% in Puruliya to 77.3% in Birbhum. Women aged 30–34 were significantly more likely to use contraception (OR = 1.47), while those aged 45–49 were less likely to use it (OR = 0.74). Women with mixed gender composition of living children (OR = 1.48) were more likely to use contraception, compared to women with no children (OR = 0.21) or daughters only (OR = 0.80). SC women (OR = 1.63) and Christians (OR = 2.17) showed higher usage. Wealthier women (OR = 1.26) and urban residents also reported higher use. Moreover, son preference continues and women married after 18 are less likely to adopt modern methods. These findings highlight the need to improve reproductive health outcomes and overcome barriers to increasing contraceptive uptake.

**Conclusion:**

Targeted interventions focusing on education, awareness-building, and improving access to diverse contraceptive options are recommended to empower women in making informed reproductive choices and advancing reproductive rights and health equity. Also, effective modern contraceptive services must overcome legal policy, social, cultural and structural barriers to benefit more women.

## Background

Providing universal access to sexual and reproductive health services, including the ability for all people to choose their preferred contraceptive methods, is a fundamental human right and a central objective of target 5.6 of the Sustainable Development Goals (SDGs). This target, supported by the United Nations, is vital for achieving the 2030 Agenda’s commitment to ensuring that ‘no one will be left behind’ [1]. In this regard, contraception is essential to enable people for making informed decisions concerning their reproductive lives and preferences for childbirth [2, 3]. As the global population reached to 8 billion in 2022 with two-thirds of this growth occurring in developing countries, it emphasizes the urgency of managing population growth through effective family planning, of which modern contraception plays an important role in this effort [4–9]. Promotion of modern contraceptive methods enhances gender equality, educational attainment, and economic empowerment for women, while also serving as an important strategy to prevent maternal mortality, reduce unsafe abortions, and reduce the spread of sexually transmitted diseases such as HIVs [10, 11]. Globally in 2019, 44% of women of reproductive age were using a modern method of contraception [12]. Between 2000 and 2020, the numbers of women using modern contraception increased from 663 million to 851 million [11, 13]. Despite this progress, women of reproductive age who are satisfied with the need for modern methods (SDG indicator 3.7.1) of family planning were 77.5% globally in 2022, a slow increase since 1990 (67%), which highlights barriers such as limited method choice, limited access to services (especially among vulnerable groups), fear of side effects, opposition from culture or religion and gender-based inequality [14, 15, 11]. In 2019, a staggering 214 million women in low- and middle-income countries did not use any contraceptive method despite wanting to avoid pregnancy [16].

India took a pioneering step in 1952 by launching the world’s first nationwide family planning program, demonstrating a longstanding commitment to creating an environment conducive to reproductive rights and informed decision-making about contraceptive use, as well as engaging in policy formulation [17]. Despite these efforts, the latest report of National Family Health Survey (NFHS-5) revealed that only 56.5% of married women aged 15–49 in India utilized any modern contraceptive method, with variations observed between urban (58.5%) and rural (55.5%) areas [17]. This disparity partly stems from prevalent misconceptions and religious taboos favoring traditional methods over modern ones [18]. Consequently, recent initiatives have focused on mitigating access barriers by enhancing the quality of family planning counseling, as evidenced by studies showing higher modern contraceptive prevalence in Southern Indian states compared to the Empowered Action Group (EAG) states, where traditional methods remain prevalent [19]. Understanding the dynamics of contraceptive acceptance requires considering multifaceted factors such as age at marriage, educational attainment, economic status, religious beliefs, media exposure, caste, and the number of living children, all of which play interconnected roles in shaping individuals’ attitudes towards family planning [19–21].

While existing research has shed light on these factors, a significant research gap is evident due to the geographical skewness in the distribution of contraceptive related studies, which are predominantly concentrated in high-focus states of India. There is a lack of research evidence on certain regions, particularly in states like West Bengal. This state characterized by its multi-ethnic population and wide-ranging socio-cultural diversity confounding with varied levels of development across its districts. This diversity contributes to a heterogeneous health status among its population [22–25]. Along this backdrop, the rate of unintended pregnancy concurrent with the higher exposure to multiple-high risk fertility behavior among currently married women and prevalent used of traditional contraceptive based family planning in West Bengal remains consistently higher than the national average of India [22] which further need to study to inform tailored health services and family planning interventions.

Therefore, this study aims to investigate the differentials in the use of modern contraceptive methods across the diverse socio-demographic settings. By concentrating on the district-specific spatial diversity within the state, the study seeks to uncover the underlying factors that influence modern contraceptive use among currently married women of reproductive age in West Bengal. This approach will provide a comprehensive understanding of how local socio-demographic dynamics shape contraceptive behavior, thereby contributing valuable insights into family planning and advanced reproductive health strategies tailored to promote health equity among women in West Bengal. Aligning with global health goals, this research specifically supports ‘Good Health and Well-being’ and ‘Gender Equality’. Thus, this study can help improve family planning outcomes, reduce disparities, and promote reproductive health and well-being in West Bengal and beyond.

## Materials and methods

### Data source

Data for this study were accessed from the DHS (Demographic Heath Surveys) program’s official database after receiving permission through an online request detailing the purpose of the study. DHS collects data through a nationally representative cross-sectional survey for countries. The Indian Demographic and Health Survey also known as National Family Health Survey (NFHS), which is the main source of data used in this study. In this study, data are restricted to currently married and non-pregnant reproductive aged women. Based on these criteria, the sample size from the fifth round of NFHS was 15,841 women in 2019-20 for the state of West Bengal.

### Study variables

In this study, modern contraceptive use is defined as the percentage of currently married non-pregnant women of reproductive age who reported using a family planning method during the survey interview. Pills, intrauterine devices (IUDs), injections, diaphragm, male condoms, female condoms, female sterilization, male sterilization, emergency contraception, foam or jelly, lactational amenorrhea method (LAM), standard days method (SDM), and other modern methods constitute modern family planning methods. Table 1 summarized the study variables, including the dependent variable and independent variables along with their respective categories.

**Table 1:** Summarize of the study variables

Variables	Categories
**Dependent variable**	
Modern contraceptive use	Yes / No
**Independent variable**	
Age of respondent	15–19, 20–24, 25–29, 30–34, 35–39, 35–39, 40–44, and 35–49
Gender composition of living children	Sons only, Daughters only, Mixed, and No living children
Age at marriage	< 18 / ≥18
Social communities	Scheduled Caste (SC), Scheduled Tribe (ST), Other Backward Classes (OBC), and Others
Religion	Hindu, Muslim, Christian, and Others
Educational status of respondent	No Education, Primary (up to 5th grade), Secondary (up to grade 10) and Higher (above secondary level)
Family planning message through media	Yes / No
Wealth quintile	Poorest, Poorer, Middle, Richer, and Richest
Place of residence	Urban / Rural

### Analytical measures

The study utilized bi-variate and multivariate analyses to explore the variations and factors influencing modern contraceptive use among currently married non-pregnant women in West Bengal. Bi-variate analysis serves to scrutinize the relationship between two chosen variables. Selected independent variables undergo cross-tabulation with the dependent variables to elucidate their one-to-one associations. The significance of these associations is assessed through the Chi-square test, which evaluates the degree of dependence between categorical variables.

Subsequently, variables demonstrating significant associations at the bi-variate level are integrated into the multivariate analysis. Multivariate logistic regression is then employed to discern the independent effects of explanatory variables on the outcomes of interest. The formula for logistic regression is:

$$\:\text{log}\left(\frac{p}{1-p}\right)={\beta\:}_{0}+{\beta\:}_{1}{X}_{1}+{\beta\:}_{2}{X}_{2}+….+{\beta\:}_{n}{X}_{n}$$

Where,

- $\:p$= probability of the event occurring
- $$\:{\beta\:}_{0}$$= intercept
- $\:{\beta\:}_{1}$, $\:{\beta\:}_{2}$,…., $\:{\beta\:}_{n}$= coefficients for the independent variables
- $\:{X}_{1}$, $\:{X}_{2}$,….,$\:{X}_{n}$=values of the independent variables.

Odds ratios (OR) and 95% confidence intervals (CI) were reported in the study to quantify the strength and direction of these effects.

## Results

### Prevalence of modern contraceptive use

**Fig. 1:**
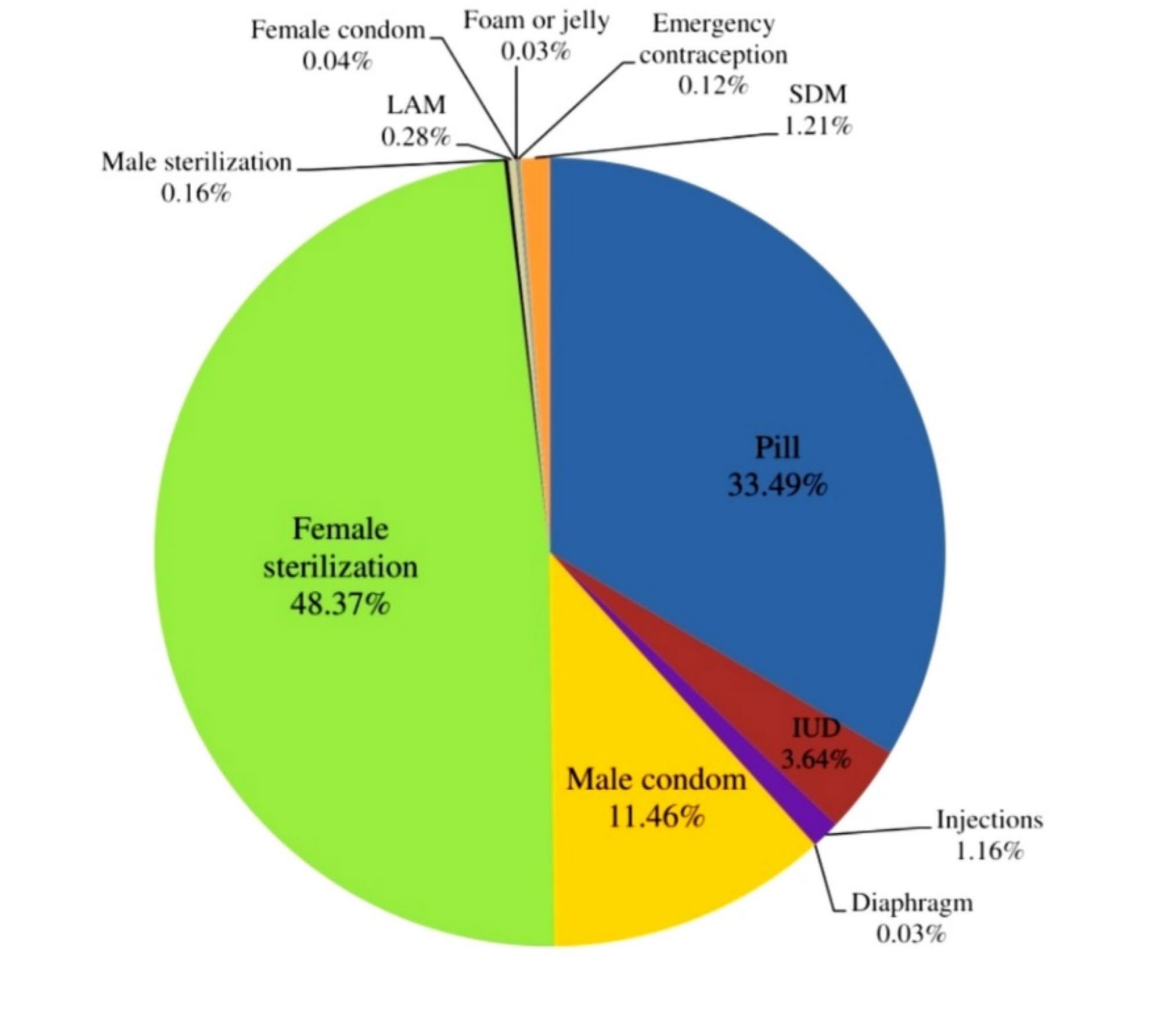
Percentage sharing of modern contraceptive use in West Bengal (2019-20)

Figure 1; Table 2 delineates the distribution of modern methods used currently married non-pregnant women in West Bengal during the period of 2019-20, segmented into rural and urban categories. The most widely used method is female sterilization, with 48.37% of women opting for this method, showing higher adoption in rural areas (50.3%) compared to urban areas (44.01%). The contraceptive pill is the second most popular method, used by 33.49% of women, with slightly higher usage in rural areas (33.7%) than in urban areas (33.03%). Male condom usage is more common in urban areas (16.52%) than in rural areas (9.23%), indicating a significant urban-rural divide in this method’s adoption. IUDs are used by 3.64% of women, slightly more in rural areas (3.82%) than urban (3.23%). Other methods such as injections, diaphragms, and female condoms have relatively low prevalence rates (below 2%), with marginal differences between rural and urban areas. Male sterilization is almost negligible at 0.16% overall, with minimal differences between rural (0.17%) and urban (0.14%) areas. Additionally, emergency contraception and methods like LAM, foam or jelly, and SDM are rarely used, with prevalence rates below 1.3% across both rural and urban settings.

**Table 2:** Prevalence rate (in %) of modern contraceptive use in rural and urban areas of West Bengal (2019-20)

Modern methods	Rural	Urban
Pill	33.7	33.03
IUD	3.82	3.23
Injections	1.12	1.26
Diaphragm	0.02	0.06
Male condom	9.23	16.52
Female sterilization	50.3	44.01
Male sterilization	0.17	0.14
LAM	0.31	0.22
Female condom	0.03	0.06
Foam or jelly	0.04	0.00
Emergency contraception	0.10	0.16
SDM	1.16	1.31

*Source: Computed from NFHS-5 dataset (2019-20)*,* West Bengal*

### District-wise discrepancy of modern contraceptive use

**Fig. 2:**
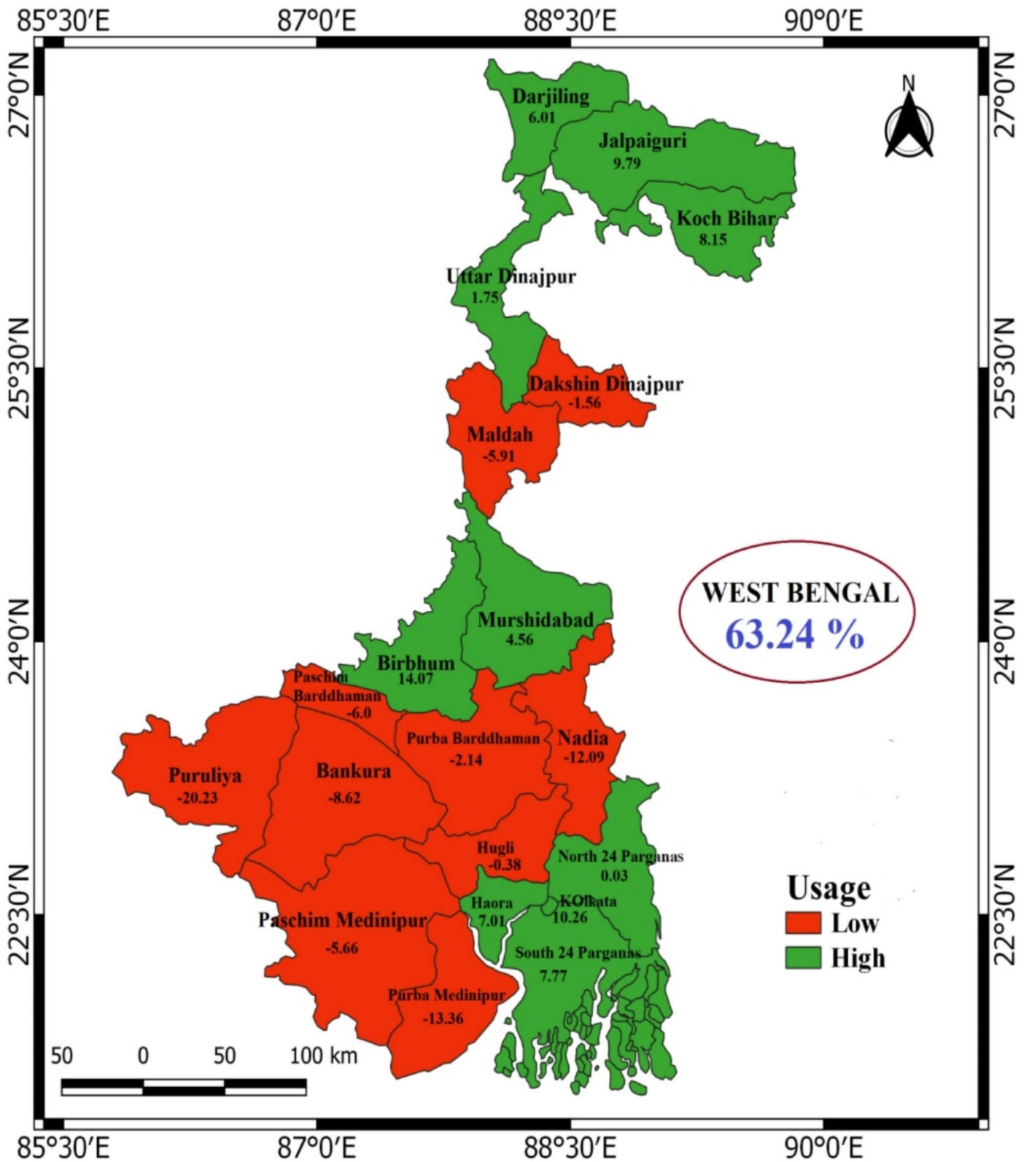
District-wise variation in modern contraceptive use in West Bengal (2019-20)

Figure 2 illustrates district-wise variation in modern contraceptive use in West Bengal. The wide range of contraceptive usage rates, from 43% in Puruliya to 77.3% in Birbhum, underscores the heterogeneous nature of contraceptive practices within the state. Districts with higher contraceptive usage rates (from min. 0.03% points to max. 14.07% points) from the baseline (state’s average), such as Birbhum, Kolkata, Jalpaiguri, Koch Bihar, Haora, North 24 Parganas, South 24 Parganas, Darjiling, Uttar Dinajpur and Murshidabad may benefit from better access to health care services, higher education levels and stronger community support for family planning initiatives, whereas districts like Dakshin Dinajur, Maldah, Puruliya, Purba Medinipur, Paschim Medinipur, Nadia, Bankura, Hugli, Purba Barddhaman and Paschim Barddhaman report lower contraceptive usage rates (from min. -0.38% points to max. -20.23% points) from the baseline.

### Differentials of modern contraceptive use

Disparities of modern contraceptive method usage (Fig. 2) reveals several significant patterns and differentials across various background characteristics (Table 3). It includes that, there is a notable association between age group and modern contraceptive use; younger women aged 15–19 exhibit the lowest prevalence of modern contraceptive use, with only 41.05% reporting usage. This percentage steadily increases with age, peaking among women aged 30–34, where 71.18% use modern contraceptives. Following this peak, there is a gradual decline in contraceptive use in the older age groups, with 64.95% among women aged 40–44 and 59.50% among those aged 45–49. Along with the presence of both sons and daughters in the family is associated with higher contraceptive usage (72.3%), suggesting a desire for family balance or completion, while those with single children either sons only (63.98%) or daughters only (59.01%) showed lower rates. Additionally, Christians showed the highest modern contraceptive usage (73.42%), followed by Hindus (63.85%), and Muslims (61.48%). Religious beliefs and norms regarding family planning practices may play a significant role in influencing these patterns. Furthermore, age at marriage, social community, education level, exposure to family planning messages through media, and wealth quintile also demonstrate statistically significant associations with contraceptive usage. For instance, women married below 18 years of age, belonging to certain social communities like SCs, having no education or primary education, not exposed to family planning messages through media, and falling within the poorest or poorer wealth quintiles demonstrate higher rates of contraceptive usage. On the other hand, women married at 18 years and above, belonging to particular communities like STs, having secondary or higher education, receiving family planning messages through media, and falling within richer or richest wealth quintiles, tend to show lower rates of modern contraceptive usage.

**Table 3:** Modern contraceptive use by socio-demographic characteristics among women in West Bengal (*N* = 15,841; 2019-20)

Background characteristics	No	Yes	χ^2^ value & sig. level	Frequency (*N*)
***Age group***				
15–19	58.95	41.05	317.52*	770
20–24	43.39	56.61		2,330
25–29	35.71	64.29		2,912
30–34	28.82	71.18		2,640
35–39	30.95	69.05		2,596
40–44	35.05	64.95		2,107
45–49	40.50	59.50		2,486
***Gender composition of living children***				
Sons only	36.02	63.98	971.6*	4,746
Daughters only	40.99	59.01		3,469
Mixed	27.74	72.26		6,413
No living children	73.70	26.30		1,213
***Age at marriage***				
Above 18	40.82	59.18	75.4*	6,752
Below 18	33.76	66.24		9,089
***Social communities***				
SC	32.08	67.92	98.6*	4,712
ST	42.05	57.95		1,089
OBC	38.51	61.49		1,893
Others	38.34	61.66		8,147
***Religion***				
Hindu	36.15	63.85	9.3*	11,622
Muslim	38.52	61.48		3,991
Christian	26.58	73.42		131
Others	38.11	61.89		97
***Education***				
No education	32.20	67.80	129.8*	3,599
Primary	31.18	68.82		3,176
Secondary	40.04	59.96		7,884
Higher	41.75	58.25		1,182
***Family planning message through media***				
No	36.28	63.72	9.5*	9,360
Yes	37.51	62.49		6,481
***Wealth quintile***				
Poorest	35.23	64.77	19.8*	5,325
Poorer	35.51	64.49		4,404
Middle	39.25	60.75		2,971
Richer	39.59	60.41		2,099
Richest	35.92	64.08		1,042
***Place of residence***				
Urban	36.86	63.14	0.17	4,447
Rural	36.71	63.29		11,394

Note: N = sample size; *p < 0.05

Source: *Computed from NFHS-5 dataset (2019-20)*,* West Bengal*

### Determinants of modern contraceptive use

The determinants influencing modern contraceptive usage in West Bengal encompass various socio-demographic factors (Table 4). Age is a crucial factor, with women aged 25–29 and 30–34 have significantly higher odds of using modern contraceptives, with odds ratios of 1.23 (95% CI: 1.00–1.52) and 1.47 (95% CI: 1.18–1.82), respectively. This indicates that women of these age groups are 23% and 47% more likely to use contraceptives compared to those aged 15–19, who serve as the reference group. However, the likelihood of contraceptive use decreases for older age groups, with women aged 45–49 being 26% less likely to use modern contraceptives (OR = 0.74, 95% CI: 0.59–0.92). Further, age at marriage influences contraceptive use, with women who married at or after 18 years being 11% less likely to use modern contraceptives compared to those who married before 18 (OR = 0.89, 95% CI: 0.81–0.96). The gender composition of living children also plays a significant role, as women with only daughters are less likely to use modern contraceptives (OR = 0.80, 95% CI: 0.72–0.88) compared to those with only sons. In contrast, women with both sons and daughters (mixed composition) are 48% more likely to use modern contraceptives (OR = 1.48, 95% CI: 1.34–1.62). Women with no living children are the least likely to use modern contraceptives, with an odds ratio of 0.21 (95% CI: 0.18–0.25), indicating a 79% lower likelihood.

Social community affiliations also significantly impact contraceptive use. SC women are 63% more likely to use modern contraceptives (OR = 1.63, 95% CI: 1.39–1.99) compared to ST women, while OBC and women from other social categories also show higher odds, with ORs of 1.32 (95% CI: 1.07–1.68) and 1.31 (95% CI: 1.13–1.52), respectively. In addition, religion also affects contraceptive use, with Christian women being over twice as likely (OR = 2.17, 95% CI: 1.25–3.17) to use modern contraceptives compared to Hindu women, while Muslim women do not show a significant difference.

Education level is another influential factor, with women who have secondary education being 23% less likely to use modern contraceptives (OR = 0.77, 95% CI: 0.68–0.87) compared to those with no education. Interestingly, exposure to family planning messages through media is associated with a reduced likelihood of using modern contraceptives (OR = 0.85, 95% CI: 0.77–0.93). Economic status, measured by wealth quintiles, shows some influence, with the richest women having a higher likelihood of using modern contraceptives (OR = 1.26, 95% CI: 0.95–1.46), though the result is marginally significant. Finally, place of residence shows a significant role influence on contraceptive use, with women in rural areas being 5% less likely to use modern contraceptives (OR = 0.95, 95% CI: 1.16–2.31) compared to those in urban areas.

**Table 4:** Odds ratios for use of modern contraceptive by their background characteristics in West Bengal, 2019–20

Background characteristics	Odds Ratio	95% confidence interval	
		Lower	Upper
***Age group***			
15–19^®^	1.00		
20–24	1.12	0.91	1.38
25–29	1.23*	1.00	1.52
30–34	1.47*	1.18	1.82
35–39	1.22	0.98	1.52
40–44	0.97	0.77	1.22
45–49	0.74*	0.59	0.92
***Gender composition of living children***			
Sons only ^®^	1.00		
Daughters only	0.80*	0.72	0.88
Mixed	1.48*	1.34	1.62
No living children	0.21*	0.18	0.25
***Age at marriage***			
< 18 ^®^	1.00		
≥ 18	0.89*	0.81	0.96
***Social communities***			
ST ^®^	1.00		
SC	1.63*	1.39	1.99
OBC	1.32*	1.07	1.68
Others	1.31*	1.13	1.52
***Religion***			
Hindu ^®^	1.00		
Muslim	0.89	0.78	1.02
Christian	2.17*	1.25	3.17
Others	1.27*	0.71	2.27
***Education***			
No education ^®^	1.00		
Primary	0.95	0.87	1.10
Secondary	0.77*	0.68	0.87
Higher	0.82	0.67	1.05
***Family planning message through media***			
No ^®^	1.00		
Yes	0.85*	0.77	0.93
***Wealth quintile***			
Poorest ^®^	1.00		
Poorer	1.05	0.94	1.15
Middle	0.91	0.78	1.04
Richer	0.98	0.82	1.16
Richest	1.26*	0.95	1.46
***Place of residence***			
Urban ^®^	1.00		
Rural	0.95*	1.16	2.31

Notes: *p < 0.05, ^®^*= Reference category*

Source: *Computed from NFHS-5 dataset (2019–20)*,* West Bengal*

## Discussion

This research uncovers crucial insights into the patterns and drivers of modern contraceptive use in West Bengal. Where, female sterilization emerges as the dominant contraceptive method in both rural and urban areas, with higher adoption in rural regions. This preference for permanent methods can be linked to factors like accessibility, cultural beliefs, and healthcare practices [26].The low uptake of male sterilization reflects entrenched gender norms, placing the contraceptive burden primarily on women [27]. In contrast, in Paschim Barddhaman district of West Bengal, IUD and ligation are most commonly adopted procedure among tribal women [28]. Another cross-sectional study conducted among 212 reproductive women in rural areas of Nadia district, West Bengal, revealed that the utilization of injectable contraceptives was inadequate due to a lack of knowledge and practice regarding this method among the participants [29]. On the contrary, while male condoms remain prominently used in contraception, their uptake is notably higher in urban areas compared to rural areas. Despite extensive awareness campaigns and easy accessibility, the usage of condoms has not seen a significant rise. Other modern methods, such as the pill, IUDs, and injections, exhibit relatively low rates of use, underscoring potential differences in accessibility or preference among populations and some previous studies have reported that these methods are not being used by women in West Bengal as lack of knowledge, indecisiveness, lack of interpersonal communication and functional knowledge are major barriers for them [30, 31]. So, this study considered that this modern method must be sincerely promoted through social marketing and the frontline health workers can play a pivotal role. Furthermore, the minimal use percentages of diaphragms, female condoms, and emergency contraceptive methods indicate limited uptake and availability of these methods in the region, emphasizing the need for increased awareness and access to a diverse range of contraceptive options.

The analysis also shows disparities in contraceptive use based on socio-demographic characteristics. Younger women, particularly those aged 15–19, exhibit the lowest usage rates. And women age beyond 34, the odds of using modern contraceptive begin to decline could be attributed to several factors, including the reduced perceived need for contraception as women approach the end of their reproductive years, as well as cultural or health-related factors that may influence contraceptive behavior among older women [32, 33]. These findings align with broader demographic research that shows contraceptive use typically peaks among women in their late twenties to early thirties, a period often associated with increased family planning needs. The decline in contraceptive use among older women is consistent with a natural decrease in fertility intentions as women age [34–38]. This pattern suggests that contraceptive use is closely tied to a women’s potentially delaying contraceptive use due to lower perceived risk or lack of knowledge.

The influence of the gender composition of living children on contraceptive use is particularly noteworthy. The significantly lower contraceptive use among women with only daughters suggests the persistence of son preference, which has been well-documented in research studies of Indian society [39]. This cultural norm may lead to continued childbearing until a son is born, delaying or reducing the use of contraceptives. In contrast, women with both sons and daughters are more likely to use contraceptives, indicating that achieving a perceived ideal family composition might prompt the adoption of family planning measures [40–44]. The very low likelihood of contraceptive use among women with no living children reflects the desire for children, which often takes precedence over family planning in such contexts [45, 46, 26].

The finding that women who married at or after 18 years are slightly less likely to use modern contraceptives compared to those who married earlier is intriguing. While early marriage is typically associated with higher fertility and lower contraceptive use [47], this study suggests that early-married women may be more inclined to adopt contraception earlier in life as they start families sooner [48]. This highlights the need for targeted family planning education and services for young married women, particularly those who marry before the legal age of 18, to ensure they have access to contraceptive options as they navigate early childbearing.

The significant differences in contraceptive use across social communities are well-documented in the literature, where marginalized groups often face barriers to accessing family planning services [49–52]. Besides, modern contraceptive methods play a crucial and cost-effective role in public health interventions for developing countries [53]. Previous research has shown that religious beliefs can either promote or hinder the adoption of family planning, depending on the doctrinal stance and community norms [54]. Christian doctrines often promote responsible family planning within the context of marriage, leading to greater acceptance of modern contraceptive methods among Christian communities [55, 56]. The lack of significant difference between Hindu and Muslim women in this study might indicate a convergence in contraceptive behavior across these religious groups, potentially due to increasing awareness and availability of family planning services [57, 58]. And also, Islam does not favor permanent family planning methods for Muslim women compared to Hindu women, who tend to prefer such kind of modern contraception like sterilization over temporary methods [49].

The inverse relationship between education and contraceptive use, particularly the lower likelihood of use among women with secondary education compared to those with no education, is somewhat unexpected. While higher education is generally associated with greater autonomy and better access to information, which can lead to higher contraceptive use [59, 60], this finding suggests that other factors, such as economic status, cultural norms, or unmet needs for family planning, may override the effects of education. This highlights the complexity of contraceptive behavior and the need for comprehensive strategies that address multiple determinants, not just educational attainment [60]. The finding that women exposed to family planning messages through media are less likely to use contraceptives is counterintuitive and suggests that these messages may not be as effective as intended. This could be due to a variety of factors, including the quality and content of the messages [61], their reach, or the presence of other barriers to contraceptive use that media exposure alone cannot overcome. Some previous literatures found that tribal women having high mass media exposure is associated with the increased modern contraceptive practice [62, 63]. This underscores the importance of designing and delivering family planning messages that are culturally sensitive, tailored to the target audience, and complemented by broader efforts to improve access to services.

The relatively small differences in contraceptive use across wealth quintiles, with the richest women being only slightly more likely to use contraceptives, suggest that economic status may not be the primary determinant of contraceptive behavior in West Bengal. This finding is consistent with other studies that have shown that while wealth can influence access to health services, other factors such as education, social norms, and geographic location may play more significant roles in shaping contraceptive use [64].

The slight difference in contraceptive use between urban and rural women, with rural women being marginally less likely to use modern contraceptives, reflects ongoing urban-rural disparities in access to healthcare and family planning services [65]. However, the narrowing of this gap suggests improvements in rural healthcare infrastructure and the increased availability of family planning services in rural areas. This is a positive development, indicating that rural women in West Bengal are increasingly able to access and use contraceptives, though continued efforts are needed to address any remaining barriers.

All in all, the study provides an accurate picture of the in-depth examination of modern methods use, uncovering trends and preferences that shed light on the cultural and social factors shaping family planning choices in West Bengal. By incorporating district-level data, the study reveals significant geographic variations in contraceptive use, enabling targeted interventions in areas with lower uptake. These insights are essential for enhancing family planning services and addressing disparities across the state.

Although the study has strength, it is not without its limitations. Its cross-sectional design restricts the ability to establish causality, as it captures data at a single point in time. Additionally, the reliance on self-reported data may introduce recall bias or social desirability bias, potentially leading to inaccuracies in reporting contraceptive use. Also, the study doesn’t thoroughly examine obstacles to adopting certain modern contraceptives, such as male sterilization and temporary methods. Moreover, the absence of qualitative insights into cultural or personal motivations behind contraceptive choices limits a fuller understanding of the underlying factors influencing family planning behaviors.

## Medical implications and recommendations

Access to contraceptive information and services is essential for safeguarding health and human rights. The study reveals a concerning trend in rural areas, where women rely heavily on permanent methods like female sterilization, limiting their access to temporary and reversible contraceptives. This not only restricts their reproductive autonomy but also exposes them to potential health risks associated with surgical procedures. Furthermore, the underutilization of male sterilization and modern temporary methods like IUDs and injections suggests a gender imbalance in family planning responsibilities, placing the burden disproportionately on women. Additionally, the low adoption of emergency contraception and underuse of methods like diaphragms and female condoms indicate significant gaps in awareness and availability, potentially leading to higher rates of unintended pregnancies and associated health risks.

To improve family planning outcomes and address the identified gaps, it is essential to promote a basket of choice of modern contraceptives. Frontline health workers, particularly in rural areas, should be trained to provide comprehensive counseling on temporary methods, including IUDs, injections, and male sterilization, to reduce the overreliance on female sterilization. Strengthening community-based programs that challenge gender norms and promote shared contraceptive responsibility between men and women can also help balance the contraceptive burden. Additionally, increasing access to and knowledge of emergency contraception and condoms especially in underserved areas, will further improve reproductive health outcomes and reduce the risk of unintended pregnancies, and side effects like HIVs and other sexually transmitted infections (STIs).

## Conclusion

The study highlights the intricate interaction of socio-demographic and cultural factors in influencing the utilization of modern contraceptives among currently married non-pregnant women in West Bengal. The most commonly used method was female sterilization, indicating a need to promote and improve access to other modern contraceptive options. In particular, the distribution of male and female condoms, IUDs emergency contraceptive pills (ECP), and *‘Chhaya’* pills should be increased [66]. It is recommended to diversify contraceptive options by actively promoting access to a variety of modern methods, thereby offering individuals a broader range of family planning options. A new intervention taken up by the Ministry of Health and Family Welfare (MoHFW), the 360-degree media campaign can create demand for contraceptives in West Bengal and its promotion is less visible in the state, which includes television advertisements, yearlong all India radio chat shows like the *‘Hum Do’* initiative, posters, and hoardings [66–68]. The MoHFW has spent a lot to boost every stakeholder with family planning mantras like *‘Plan Bante Hai’* (Let’s make a plan), *‘Achchi Adat Hai’* (It’s a good practice) etc [68]. Besides, the multimedia campaign includes WhatsApp messages, a dedicated website for family planning [69] and a toll free helpline number (1800 116 555) for providing family planning advices, about which women in West Bengal may not be aware or don’t give any importance. Further, accredited social health activist (ASHA) workers should subtly emphasize the home delivery of modern contraceptives to beneficiaries and prioritize the development of their other responsibilities. It is also essential to improve the skills of doctors, nurses, and midwives through training and professional development, so that they can provide effective family-centered counseling to all women who need it. Accreditation of more private/NGO facilities is required in West Bengal to increase the provider base for family planning services through public-private partnership (PPP). Furthermore, the promotion of modern contraception can be achieved by displaying posters and utilizing audio and video materials in various educational institutions.

In addition, there is a need to strengthen outreach and inter-personal communication efforts, with a particular focus on informing individuals about the dynamic nature of available contraception and family planning policies. With this, targeted interventions focusing on education, awareness-building, and improving access to a diverse range of contraceptive options are essential for empowering women to make informed choices about their reproductive health.

## Data Availability

The dataset used in this study is publicly available from the Demographic Health Survey (DHS) website (https://dhsprogram.com) and could be accessed upon a data request subject to non-profit and academic interest only. The dataset modified for use in this paper is available upon reasonable request to the corresponding author.
